# The performance of growth charts in well term newborns in screening for hypoglycemia

**DOI:** 10.1038/s41372-025-02373-3

**Published:** 2025-08-05

**Authors:** Marwa A. Khalil, Samantha D’Aversa, Tara Lozy, Nicole T. Spillane

**Affiliations:** 1 Department of Pediatrics, Division of Neonatology, Hackensack University Medical Center, Hackensack, NJ USA; 2 Hackensack Meridian School of Medicine, Nutley, NJ USA; 3 Department of Pediatrics, Hackensack University Medical Center, Hackensack, NJ USA

**Keywords:** Outcomes research, Paediatrics

## Abstract

**Objective:**

To compare the performance of Fenton Growth Chart (FGC) with WHO Growth Chart (WGC) to identify term infants at risk for hypoglycemia.

**Study design:**

A retrospective study of infants screened for hypoglycemia due to SGA or LGA status determined by FGC and/or WGC.

**Results:**

Nine hundred and seventy infants were included. There was 47.7% agreement between growth charts. A total of 283 (29.2%) newborns developed hypoglycemia. Of those with hypoglycemia, 53.7% were identified by both charts. WGC was more sensitive for all categories of hypoglycemia examined; hypoglycemia (81.3% [CI 76.2–86.7] vs 72.4% [CI 66.8–77.6]), severe hypoglycemia (80.8% [CI 67.5–90.4] vs 71.2% [CI 56.9–82.9]), and hypoglycemia requiring NICU transfer (88.5% [CI 69.9–97.6] vs 65.4% [CI 44.2–82.8). The FGC was more specific for all hypoglycemia categories.

**Conclusions:**

There was poor agreement between WGC and FGC for hypoglycemia in term infants. The WGC was more sensitive in detecting hypoglycemia and was non-inferior to the FGC.

## Introduction

Measurement of birth weight is a valuable tool in assessing newborn wellness. Infants classified as small-for-gestational-age (SGA; birth weight <10th percentile for gestational age and sex) or large-for-gestational-age (LGA; birth weight >90th percentile for gestational age and sex) are at a greater risk for neonatal complications as compared to their appropriate-for-gestational age counterparts. Some complications include neonatal hypoglycemia, hyperbilirubinemia, respiratory distress, and NICU admission [1–5]. Neonates with risk factors undergo screening as clinical signs of hypoglycemia may be subtle and mimic the manifestations of other conditions. Undetected low blood glucose may result in serious morbidity including cyanotic episodes, seizures, apneic or tachypneic spells, feeding difficulties, and long-term adverse neurodevelopmental outcomes [6, 7]. Thus, it is essential to have an accurate tool to categorize infants at risk for neonatal hypoglycemia due to growth classification as SGA or LGA.

Many growth standards exist for the assessment of birth weight. These standards are based on diverse populations which vary by ethnicity, country, type of feeding, and gestational age [8]. The World Health Organization (WGC) and Fenton Preterm Growth Charts (FGC) are two commonly used growth standards for the term population. The WGC is based on the Multicentre Growth Reference Study of term infants born between 37–42 weeks in which all gestational ages were combined [9]. Meanwhile, the FGC is based on fetal growth beginning at 22 weeks and includes changes in growth at term gestation [10, 11]. It is unclear which growth standard performs best in identifying risk for growth-related complications in the term well population [8, 12].

Due to the differences in growth classification, term infants evaluated with both curves are not consistently categorized as SGA or LGA. For the term infant, it is unclear which growth standard is most efficacious in determining the risk for hypoglycemia. This ambiguity has resulted in variability in weight-based screening criteria with discordant interfacility screening rates. A range of 7.7–15.5% of infants qualify for screening solely based on different weight-based screening guidelines [13]. The underscreening of infants truly at risk for neonatal hypoglycemia may have significant consequences, such as seizures and neurodevelopmental impairment [5, 6, 14]. In contrast, the over screening of infants may lead to unnecessary testing, separation of the infant/mother dyad, increasing medical costs, and exposure to unindicated painful procedures [14].

The goal of this study was to assess and compare the performances of the WGC and FGC to identify risk for hypoglycemia in term well newborns. Considering that well baby nurseries (WBN) internationally routinely screen infants for hypoglycemia due to SGA/LGA status, this study may have widespread impacts on WBN care practices.

## Materials/subjects and methods

**Fig. 1:**
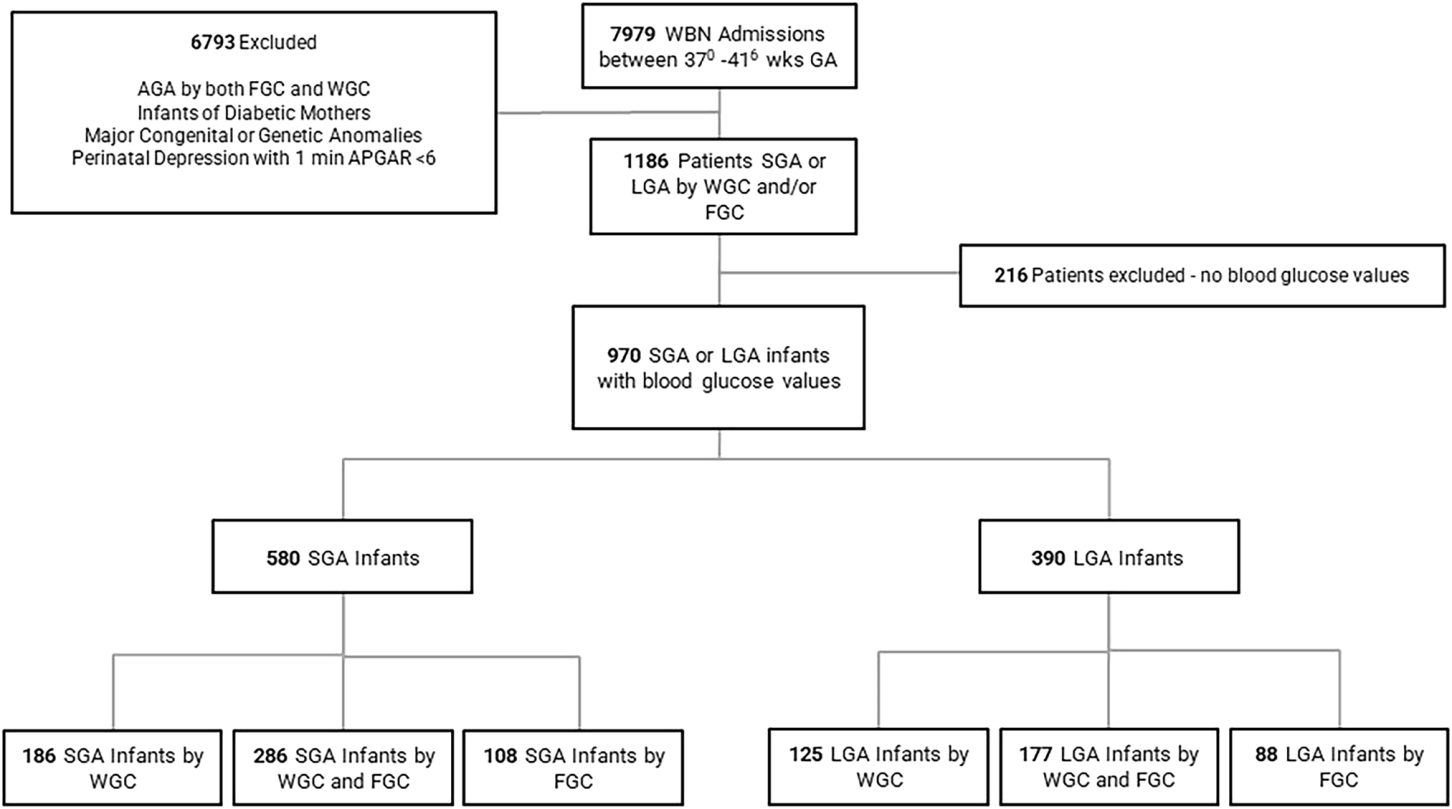
Flow chart of included and excluded study participants. WBN well baby nursery, SGA small for gestational age, LGA large for gestational age, AGA appropriate for gestational age, FGC Fenton Growth Chart, WGC WHO Growth Chart.

**Fig. 2:**
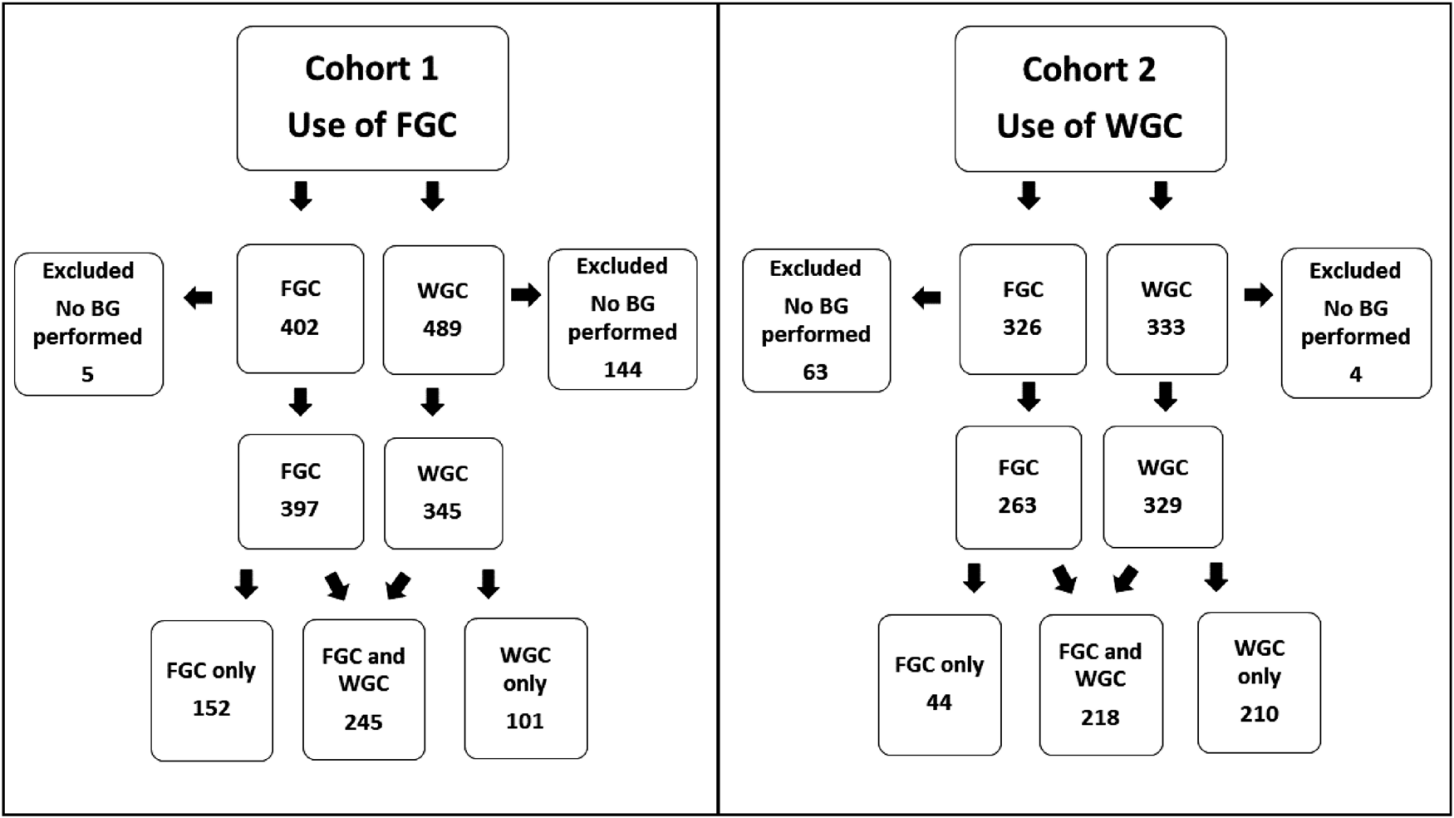
Comparison of study participants included from Cohort 1 versus Cohort 2. Cohort 1 was the time period when the Fenton Growth Chart (FGC) was utilized for growth classification. Cohort 2 was the time period when the WHO Growth Chart (WGC) was utilized for growth classification. SGA small for gestational age, LGA large for gestational age.

This is a retrospective study performed at Joseph M. Sanzari Children’s Hospital at Hackensack University Medical Center. The study population included WBN admissions born at 37 + 0 to 41 + 6 weeks between July 2021 and August 2023 whose only risk factor for hypoglycemia was SGA or LGA growth classification. During the study period, the WBN transitioned from using the FGC to the WGC for growth classification for term infants. This change impacted the population being screened for hypoglycemia due to SGA or LGA status. Between July 2021 to June 2022 (cohort 1), the FGC was used and between September 2022 to August 2023 (cohort 2) the WGC was used. The transition period, July to August 2022, was not included in the study. Newborns in both cohorts were classified by the research team as SGA, AGA or LGA using both growth charts. Exact weights corresponding to SGA and LGA for gestational age and sex were obtained from the Fenton 2013 Growth Calculator Tool [15] for classification for FGC. Similarly, exact weights corresponding to SGA and LGA for male and female term infants were obtained from WHO Growth Charts [16]. Those classified by SGA or LGA by either growth chart and had blood glucoses performed were included in the study (Figs. 1 and 2).

**Fig. 3:**
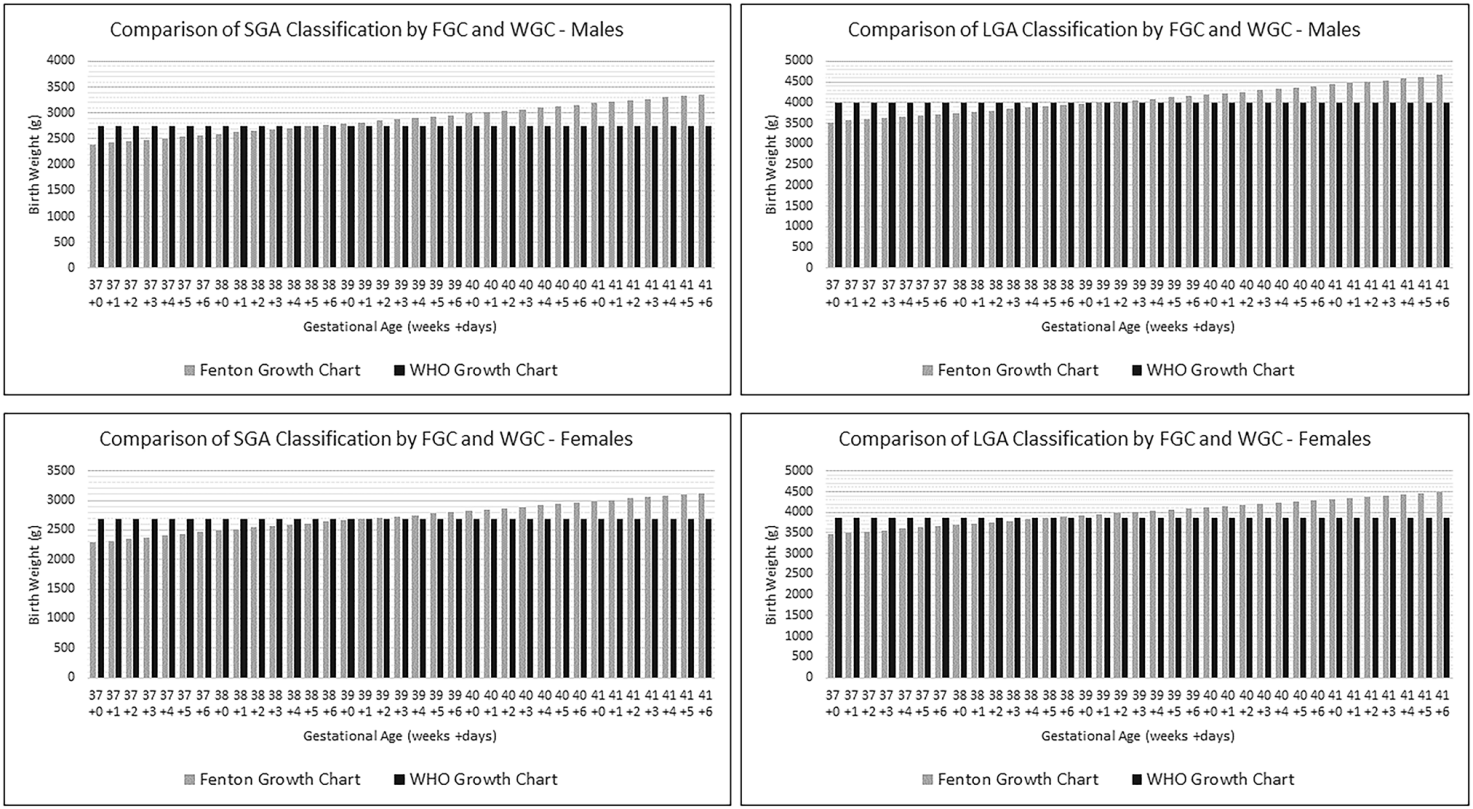
Comparison of growth classification by Fenton growth chart vs WHO Growth Chart. None.

Data were collected from electronic medical records. Information about pregnancy, labor & delivery, and neonatal nursery courses were abstracted. Infants classified as SGA or LGA by the FGC [11] or WGC [9] were included. For growth classification by the FGC, gestational age in weeks and days was utilized. The differential classification of growth categories by WGC and FGC is demonstrated in Fig. 3. Exclusion criteria included: maternal diabetes, major chromosomal or congenital anomalies, family history of persistent hypoglycemia or metabolic disorders, and perinatal depression defined as APGAR score ≤5 at one minute of life or need for advanced resuscitation. SGA or LGA infants without point-of-care blood glucose were also excluded.

There were 7979 admissions to the WBN with 4292 from July 2021 to June 2022 (cohort 1) and 3687 from September 2022 to August 2023 (cohort 2) (Fig. 1). Gestational age was assigned using the best obstetrical estimate based on the last menstrual period and confirmed using first-trimester ultrasound biometry when available. Blood glucose monitoring in the WBN was performed per unit policy (Supplemental Fig. 1). Blood glucoses were tested with Accu-Chek Inform II glucometer and reagent strips (Roche, Indianapolis, IN). Blood glucoses were monitored until 3 consecutive point-of-care (POC) levels were ≥45 and for a minimum of 24 h for SGA infants and 12 h for LGA infants. For asymptomatic infants in the first 4 hours of life, infants with any BG ≤ 25 mg/dl were immediately transferred to the NICU. After 4 hours of life, infants with any blood glucose ≤35 mg/dl were immediately transferred to the NICU. Up to two glucose gels could be administered accompanied by oral feeding in the WBN. Any infant with symptoms of hypoglycemia with a blood glucose <40 mg/dl were transferred to the NICU.

In our study, hypoglycemia was defined as a blood glucose <45 mg/dL and severe hypoglycemia was defined as <35 mg/dL. Hypoglycemia requiring intervention with glucose gel or transfer to the NICU was also reported. Hypoglycemia as an indication for NICU transfer was abstracted from the NICU admission note.

Continuous variables were summarized using means and standard deviations or medians and interquartile ranges based on the parametric status of the variable. Normality was assessed using the Shapiro–Wilk test. Categorical variables are presented as absolute counts and percentages. To assess the classification ability of the FGC and WGC growth curves for identifying patients with hypoglycemia, sensitivity, specificity, positive predictive value (PPV) and negative predictive value (NPV) were calculated. Relative values for all classification metrics (ratios of FGC to WGC values) were determined along with their 95% confidence intervals. Non-inferiority study design was selected because historically the FGC was the standard in the well nursery. Non-inferiority of the WGC compared to the FGC was concluded if the upper bound of the 95% confidence interval for the relative PPV, NPV, sensitivity or specificity did not exceed the non-inferiority margin of 1.33. This margin was pre-specified based on historical data and a threshold of 25% of the lower confidence interval [17]. All statistical analyses were performed using JMP®, Version 17.2. SAS Institute Inc, Cary, NC,1989–2024.

## Results

During the study period, there were 7979 term WBN admissions. All newborns in both cohorts were classified as SGA, AGA or LGA using both growth charts. One thousand one hundred eighty-six infants were classified as SGA or LGA by either growth chart with 216 excluded due to missing blood glucose values. The final study population consisted of 970 infants with 580 SGA infants and 390 LGA infants. IDM infants were excluded correlating with a smaller number of LGA infants. The SGA population was 186 infants classified by WGC only, 108 by FGC only and 286 by both growth charts. Correspondingly, the WGC identified 81.4% while the FGC identified 67.9% of the total SGA population. The LGA population was 125 infants classified by WGC only, 88 by FGC only, and 177 by both growth charts (Fig. 1). This corresponded to the WGC identifying 77.4% and the FGC identifying 67.9% of the total LGA population. Despite using FGC as the standard during cohort 1, 101 infants (20.3% of cohort 1) classified as SGA/LGA by WGC only had blood glucoses performed and were included. While during cohort 2 when WGC was the standard, 44 infants (9.3% of cohort 2) classified as SGA/LGA by FGC only had blood glucoses performed and were included in the study population (Fig. 2). Maternal and neonatal characteristics were analyzed for the entire patient population and for FGC and WGC subgroups. Overall, the median GA was 39 weeks (IQR 37-39w) with a median birth weight of 2755 g (IQR 2560-4025 g). Correlating with the disparity in birth weights of SGA and LGA infants, there was a bimodal distribution of birth weights in the entire study population (Supplementary Figure 2). Gestational age, birth weight, sex, and delivery/cord complications were significantly different for growth classification groups. WGC infants were younger and smaller while FGC infants were more frequently male and experienced more cord and delivery complications. As anticipated for a low-risk well nursery population, most infants were born by vaginal delivery (61.3%) with clear fluid (84.4%) and without delivery complications (91%) (Table 1).

**Table 1:** Comparison of characteristics of infants characterized as SGA or LGA by Fenton and WHO Growth Charts.

	SGA/LGA by FGC only (*n* = 196)	SGA/LGA by WGC only (*n* = 311)	SGA/LGA by both growth charts (*n* = 463)	All SGA/LGA infants (*n* = 970)	*p*
Maternal characteristics					
Maternal age, mean (SD)	32.1 (5.4)	32.3 (5.5)	32.6 (5.3)	32.4 (5.4)	0.43
Maternal Race					
Asian, *n* (%)	32 (16.6)	34 (11.1)	96 (21.1)	162 (17.0)	**<0.005**
Black, *n* (%)	10 (5.2)	23 (7.5)	27 (5.9)	60 (6.3)	0.52
White, *n* (%)	73 (37.8)	110 (36.1)	163 (35.8)	346 (36.3)	0.07
Other, *n* (%)	78 (40.4)	138 (45.2)	169 (37.1)	385 (40.4)	0.08
Ethnicity - Hispanic, *n* (%)	78 (40.8)	129 (42.2)	163 (36.1)	370 (39.0)	0.21
BMI, median (IQR)	30.7 (27.1–35.3)	30.8 (27.3–34.1)	30 (27.1–34.3)	30.4 (27.1–34.4)	0.52
Parity, median (IQR)	1 (1–2)	2 (1–3)	1 (1–2)	1 (1–2)	0.13
Gravidity, median (IQR)	2 (1–3)	2 (1–4)	2 (1–3)	2 (1–3)	0.09
Neonate characteristics					
Gestational age (weeks), median (IQR)	39 (38–40)	38 (37–40)	39 (38–39)	39 (37–39)	**<0.001**
Birthweight (grams), median (IQR)	3096 (2835–3940)	2705 (2570–3950)	2643 (2450–4069)	2755 (2560–4025)	**<0.001**
Gender - Male, *n* (%)	151 (77.4)	134 (43.1)	224 (48.3)	509 (52.5)	**<0.001**
APGAR (1 min), median (IQR)	9 (9–9)	9 (9–9)	9 (9–9)	9 (9–9)	0.40
APGAR (5 min), median (IQR)	9 (9–9)	9 (9–9)	9 (9–9)	9 (9–9)	0.68
Delivery type - vaginal, *n* (%)	124 (64.6)	177 (57.5)	287 (62.5)	588 (61.3)	0.22
Clear Amniotic Fluid Color, *n* (%)	162 (83.5)	256 (83.1)	393 (85.6)	811 (84.4)	0.60
Delivery complications - Yes, *n* (%)	25 (13.4)	26 (9.1)	31 (7.1)	82 (9.0)	**0.04**
Cord complications - Yes, *n* (%)	74 (37.9)	68 (21.9)	158 (34.1)	300 (30.9)	**<0.001**
Blood sugars (No.), median (IQR)	5 (4–8)	6 (4–8)	8 (4–9)	7 (4–8)	**<0.001**
NICU Stay - Yes, *n* (%)	3 (1.5)	9 (2.9)	14 (3.0)	26 (2.7)	0.54

Statistically significant *p*-values are in bold.

Delivery complications included: retained placenta, placenta previa, postpartum hemorrhage, shoulder dystocia and other. Cord complications included: true knot in cord, nuchal cord, cord around the body, other.

The incidence of hypoglycemia was 29.2% (283/970). Of those with hypoglycemia, 53.7% were identified for screening by both charts. Of the remaining 131 patients with hypoglycemia, 59.9% were identified by WGC compared with 40.9% by FGC. Overall, the two growth charts had 47.7% agreement. For SGA and LGA infants, there was 49.3% and 45.4% agreement between the charts, respectively. The median number of BG tests completed was 7 (IQR 4–8) for the entire study population. Infants identified for screening by both growth charts had the most blood glucoses performed. 6.1% (59/970) were transferred to the NICU with 44% (26/59) transferred for management of hypoglycemia (Table 1).

The baseline characteristics during cohort 1 and cohort 2 were also compared. In cohort 1 (FGC used), 149 infants were excluded due to missing blood glucose values while in cohort 2 (WGC used), 67 infants were excluded due to missing blood glucose values. The characteristics of cohort 1 and cohort 2 significantly differed for gestational age (39w [38-39] vs 38w [37-39], p < 0.01) and birth weight (2866 g [2580-4050] vs 2715 g [2536-3969] with younger and smaller infants in cohort 2. Cohort 2 also had more blood glucoses performed (49.4% vs 37.6% with >7 blood glucoses) and a larger proportion of black infants (Table 2).

**Table 2:** Comparison of Characteristics of Cohort 1 (FGC as standard) vs Cohort 2 (WGC as standard).

	Cohort 1 (*n* = 498)	Cohort 2 (*n* = 472)	All SGA/LGA infants from Cohorts 1& 2 (*n* = 970)	*p*
Maternal characteristics				
Maternal age, mean (SD)	32.6 (5.3)	32.2 (5.5)	32.4 (5.4)	0.27
Maternal race				
Asian, *n* (%)	90 (18.4)	72 (15.5)	162 (17.0)	0.06
Black, *n* (%)	23 (4.7)	37 (8.0)	60 (6.3)	**0.03**
White, *n* (%)	178 (36.5)	168 (36.1)	346 (36.3)	0.41
Other, n (%)	197 (40.4)	188 (40.4)	385 (40.4)	0.45
Ethnicity - Hispanic, *n* (%)	193 (39.7)	177 (38.3)	370 (39.0)	0.66
BMI, median (IQR)	30.3 (27.1–34.5)	30.4 (27.2–34.4)	30.4 (27.1–34.4)	0.93
Parity, median (IQR)	1 (1–2)	1 (1–2)	1 (1–2)	0.72
Gravidity, median (IQR)	2 (1–3)	2 (1–3)	2 (1–3)	0.51
Neonate characteristics				
Gestational age (weeks), median (IQR)	39 (38–39)	38 (37–39)	39 (37–39)	**<0.01**
Birthweight (grams), median (IQR)	2866 (2580–4050)	2715 (2536–3969)	2755 (2560–4025)	**<0.01**
Gender - Male, *n* (%)	275 (55.2)	234 (49.6)	509 (52.5)	0.08
APGAR (1 min), median (IQR)	9 (9–9)	9 (9–9)	9 (9–9)	0.94
APGAR (5 min), median (IQR)	9 (9–9)	9 (9–9)	9 (9–9)	0.62
Delivery Type - Vaginal, *n* (%)	305 (62.2)	283 (60.3)	588 (61.3)	0.55
Clear amniotic fluid color, *n* (%)	419 (84.7)	392 (84.1)	811 (84.4)	0.82
Delivery complications - Yes, *n* (%)	48 (10.3)	34 (7.6)	82 (9.0)	0.15
Cord complications - Yes, *n* (%)	158 (31.7)	142 (30.1)	300 (30.9)	0.58
Blood Sugars (No.), median (IQR)	7 (4–8)	7 (4–8)	7 (4–8)	**0.01**
NICU stay - yes, *n* (%)	13 (2.6)	13 (2.8)	26 (2.7)	0.89

Statistically significant *p*-values are in bold.

Delivery complications included: retained placenta, placenta previa, postpartum hemorrhage, shoulder dystocia and other. Cord complications included: true knot in cord, nuchal cord, cord around the body, other.

The performances of FGC and WGC to identify infants at risk for hypoglycemia, severe hypoglycemia, and hypoglycemia requiring management with glucose gel and/or transfer to the NICU were evaluated (Table 3). The sensitivity of WGC was consistently better for all the studied subcategories with sensitivities of 81.3 vs 72.4% for hypoglycemia, 80.8 vs 71.2% for severe hypoglycemia, 83.4 vs 74.9% for glucose gel, and 88.5 vs 65.4% for NICU transfer for hypoglycemia. Overall, the specificity was poor for both growth charts. However, the specificities of FGC were higher for all the studied subcategories (Table 3). Correspondingly, the relative sensitivities of the WGC were significantly better for hypoglycemia [0.89, IQR 0.88-0.90], severe hypoglycemia [0.88, IQR 0.84-0.92], need for glucose gel [0.90, IQR 0.88-0.91] and NICU transfer for hypoglycemia [0.74, IQR 0.63-0.85] while the relative specificities of the FGC were significantly better for hypoglycemia [1.64, IQR 1.57-1.72], severe hypoglycemia [1.59, IQR 1.55-1.66], need for glucose gel [1.60, IQR 1.55-1.67] and NICU transfer for hypoglycemia [1.58, IQR 1.52-1.63]). Overall, the PPV was poor but similar. The NPV for severe hypoglycemia and hypoglycemia requiring NICU transfer for both charts were reasonably good (>90%) and similar. The relative PPV and NPV trended towards WGC performing significantly better for NICU transfer, 0.87 (IQ 0.76-1.0) and 0.99 (0.99-1.00) respectively (Table 2). For the subpopulation of SGA infants, results were similar to that of the entire cohort, with improved relative sensitivity but worse relative specificity of the WGC. The relative NPV of the WGC for hypoglycemia, glucose gel and NICU transfer were better for SGA newborns while the relative PPV for NICU transfer was worse. The results for the LGA subpopulation were different. The relative sensitivities of the WGC and FGC were similar but the relative specificity of the FGC was better for all subcategories of hypoglycemia (Table 3).

**Table 3:** Comparison of the Performances of WHO and Fenton Growth Charts.

	Hypoglycemia		Severe hypoglycemia		Glucose gel		Transfer to NICU for hypoglycemia	
	FGC	WGC	FGC	WGC	FGC	WGC	FGC	WGC
All patients								
Sensitivity	72.4 [66.8, 77.6]	81.3 [76.2, 86.7]	71.2 [56.9, 82.9]	80.8 [67.5, 90.4]	74.9 [68.0, 80.9]	83.4 [77.3, 88.5]	65.4 [44.3, 82.8]	88.5 [69.9, 97.6]
Specificity	33.9 [30.4, 37.6]	20.7 [17.7, 23.9]	32.2 [29.2, 35.4]	20.2 [17.6, 22.9]	33.7 [30.4, 37.2]	21.0 [18.2, 24.0]	32.0 [29.0, 35.1]	20.3 [17.8, 23.1]
PPV	31.1 [29.2, 33.1]	29.7 [28.3, 31.1]	5.6 [4.7, 6.6]	5.4 [4.8, 6.2]	21.2 [19.7, 22.9]	20.1 [19.0, 21.3]	2.58 [1.96, 3.40]	2.97 [2.58, 3.41]
NPV	74.9 [70.7, 78.8]	72.8 [66.9, 78.1]	95.2 [92.7, 96.8]	94.9 [91.3, 97.0]	84.9 [81.2, 88.0]	84.1 [78.9, 88.2]	97.1 [95.2, 98.3]	98.5 [95.6, 99.5]
Relative Sensitivity	0.89 [0.88, 0.90]*		0.88 [0.84, 0.92]*		0.90 [0.88, 0.91]*		0.74 [0.63, 0.85]*	
Relative specificity	1.64 [1.57, 1.72]*		1.59 [1.55, 1.66]*		1.60 [1.55, 1.67]*		1.58 [1.52, 1.63]*	
Relative PPV	1.05 [1.03, 1.06]		1.04 [0.98, 1.06]		1.06 [1.04, 1.08]		0.87 [0.76, 1.00]	
Relative NPV	1.03 [1.01, 1.06]		1.00 [1.00, 1.02]		1.01 [1.00, 1.03]		0.99 [0.99, 1.00]	
SGA patients								
Sensitivity	71.0 [64.8, 77.2]	84.5 [79.6, 89.5]	70.7 [56.8, 84.7]	82.9 [71.4, 94.4]	72.9 [65.5, 80.2]	85.7 [80.0, 91.5]	58.8 [35.4, 82.2]	94.1 [82.9, 100.0]
Specificity	33.8 [29.0, 38.6]	20.1 [16.0, 24.2]	32.3 [28.3, 36.2]	18.6 [15.3, 21.8]	33.6 [29.2, 38.1]	19.8 [16.1, 23.5]	31.8 [27.9, 35.6]	18.8 [15.6, 22.1]
PPV	37.3 [32.5, 42.1]	37.0 [32.6, 41.3]	7.4 [4.8, 9.9]	7.2 [4.9, 9.5]	25.9 [21.6, 30.2]	25.4 [21.4, 29.3]	2.5 [1.0, 4.1]	3.4 [1.8, 5.0]
NPV	67.7 [61.0, 74.5]	70.1 [61.4, 78.8]	93.5 [90.0, 97.1]	93.5 [88.8, 98.1]	79.6 [73.8, 85.4]	81.3 [73.9, 88.7]	96.2 [93.5, 99.0]	99.1 [97.2, 100.0]
Relative sensitivity	0.84 [0.81, 0.86]*		0.85 [0.80, 0.90]*		0.85 [0.82, 0.88]*		0.62 [0.43, 0.82]*	
Relative specificity	1.68 [1.60, 1.81]*		1.74 [1.66, 1.85]*		1.70 [1.62, 1.81]*		1.69 [1.61, 1.79]*	
Relative PPV	1.01 [1.00, 1.02]		1.03 [0.98, 1.04]		1.02 [1.01, 1.03]		0.74 [0.56, 0.82]*	
Relative NPV	0.97 [0.95, 0.99]*		1.00 [0.99, 1.01]		0.98 [0.96, 0.99]*		0.97 [0.96, 0.99]*	
LGA patients								
Sensitivity	76.3 [66.8, 85.9]	72.3 [62.3, 82.4]	72.7 [46.4, 99.0]	72.7 [46.4, 99.0]	80.9 [69.6, 92.1]	76.6 [64.5, 88.7]	77.8 [50.6, 100.0]	77.8 [50.6, 100.0]
Specificity	34.1 [28.8, 39.3]	21.3 [16.8, 25.9]	32.2 [27.5, 36.9]	22.4 [18.2, 26.6]	33.8 [28.8, 38.8]	22.4 [18.0, 26.9]	32.3 [27.6, 37.0]	22.6 [18.4, 26.8]
PPV	21.9 [16.9, 26.9]	18.2 [13.9, 22.6]	3.0 [1.0, 5.1]	2.6 [0.8, 4.5]	14.3 [10.1, 18.6]	11.9 [8.3, 15.6]	2.6 [0.7, 4.6]	2.3 [0.6, 4.0]
NPV	85.6 [79.4, 91.8]	76.1 [67.2, 85.0]	97.6 [94.9, 100.0]	96.6 [92.8, 100.0]	92.8 [88.3, 97.3]	87.5 [80.6, 94.4]	98.4 [96.2, 100.0]	97.7 [94.6, 100.0]
Relative sensitivity	1.06 [1.04, 1.07]		1.00 [1.00, 1.00]		1.06 [1.04, 1.08]		1.00 [1.00, 1.00]	
Relative specificity	1.60 [1.52, 1.71]*		1.44 [1.39, 1.51]*		1.51 [1.45, 1.60]*		1.43 [1.38, 1.50]*	
Relative PPV	1.20 [1.19, 1.22]		1.15 [1.13, 1.25]		1.20 [1.19, 1.22]		1.14 [1.14, 1.14]	
Relative NPV	1.12 [1.08, 1.18]		1.01 [1.00, 1.02]		1.06 [1.03, 1.10]		1.01 [1.00, 1.02]	

Hypoglycemia is defined as blood glucose <45 mg/dl, severe hypoglycemia is defined as <35 mg/dl, glucose gel provided per unit guidelines, and hypoglycemia as an indication for NICU transfer obtained from NICU admission note.

*Statistically significant.

## Discussion

Screening for hypoglycemia in well neonates with risk factors is widely accepted and practiced [18]. One risk factor for hypoglycemia is deviation in growth parameters, routinely defined as <10% (SGA) or >90% (LGA) for sex. Various growth charts are utilized in the term well nursery population; however, it is unclear which one is the most sensitive and specific to identify infants at risk for hypoglycemia. Each chart is derived from a unique population leading to variability in the classification of SGA and LGA and discrepancies in screening. It is crucial to identify neonates reliably and accurately at risk for hypoglycemia due to growth disturbances.

To our knowledge, this study is the first examination of the performance of two commonly used growth charts to identify term infants at risk for hypoglycemia. Close to 1000 term infants had their growth classified using both FGC and WGC and the performance of these growth standards compared for hypoglycemia detection. We found that the WGC is more sensitive in identifying term well infants at risk for hypoglycemia compared to the FGC. The relative sensitivities of the WGC to detect hypoglycemia, severe hypoglycemia and hypoglycemia requiring intervention were all significantly better than FGC. In subgroup analyses, the WGC had significantly better relative sensitivities and relative NPV for SGA infants. Based on prespecified criteria, the WGC was non-inferior to the FGC for relative specificity, PPV and NPV. Despite controversies in defining clinically significant “hypoglycemia,” there is agreement that at risk infants benefit from screening [6, 18, 19]. WGC better identified infants at risk for hypoglycemia with its best performance for persistent/severe hypoglycemia requiring NICU transfer. Detecting severe neonatal hypoglycemia is of particular importance due to its association with worse neurodevelopmental outcomes [5, 20, 21].

Infants who are SGA/LGA have been determined to be at increased risk for complications [1–3]. These results have been confounded by heterogeneity in how infants were classified as SGA/LGA [8, 22–25]. Previous researchers have not directly compared growth standards and risk for hypoglycemia in a term population with no other risk factors for hypoglycemia. In other populations, growth charts have been shown to be inconsistent in predicting neonatal complications [26]. Reports of the incidence of hypoglycemia in term SGA/LGA infants have ranged significantly. Early studies in which hypoglycemia risk was determined by weight-for-length reported hypoglycemia incidence between 6-25%. While contemporary studies incorporating absolute weights and gestational age-and sex-specific growth report an incidence of hypoglycemia between 37 and 53% [27, 28]. The high rate of hypoglycemia (29.2%) in our cohort approximate contemporary cohorts and support the advantage of sex and gestational age-specific standards in determining hypoglycemia risk.

Additionally, in this study, we observed a significant lack of agreement in growth classification between the two charts. Only 47.7% of infants were classified as SGA and/or LGA by both growth standards. The WGC SGA/LGA cohort was smaller (2705 g vs 3096 g) and younger (38w vs 39w) than the FGC cohort. This variability in classification could lead to harm, with some infants experiencing undetected hypoglycemia while others are subject to unnecessary screenings. There is a lack of consensus on best practice in determining hypoglycemia risk based on weight. Some centers use absolute weight cut-offs, while others use weight-for-age-for-sex or a combination of the two [13, 28]. In a large multi-site study of hypoglycemia, there was no uniform screening guideline. Three unique guidelines were included in this study reflecting variability in local practice at participating sites. The heterogeneity in practice among centers resulted in significant differences in the percent of infants qualified for screening, ranging from 7.7% to 15.5% [13]. Our change in practice from the FGC to the WGC reflected uncertainty about the optimal classification system in the neonatal/pediatric community. Variability in practice results in significant discrepancies in screening practices with unknown impacts.

The incidence of hypoglycemia in healthy babies is 5–39% with no specific plasma/blood glucose values that definitively diagnoses clinically significant hypoglycemia [13, 28–32]. The accepted practice for diagnosing and treating hypoglycemia in the well nursery is POC blood glucose due to its ease of use and rapid results. However, this testing modality is inaccurate, particularly at lower glucose levels [29]. Parental anxiety, cascades of medical interventions, decreased exclusive breastfeeding, and unnecessary NICU admission are all potential negative consequences of increased screening [14]. Despite these concerns, having a highly sensitive screening test is paramount. Hypoglycemic injury and morbidities do occur and cannot be predicted based on specific clinical symptoms [5, 6, 29, 31]. The WGC offers the advantage of increased sensitivity in detecting hypoglycemia. Heightened sensitivity is particularly important for screening tests, allowing for early identification and subsequent intervention.

Our study is limited by several factors. For one, not all infants classified as SGA/LGA had blood glucoses performed. Eighteen percent of eligible infants were missing blood glucose values. The missing data may have unpredictably biased the results. Additionally, there is a potential for misclassification bias as the growth chart in use dictated the blood glucose measurements. Another limitation is the lack of information about why blood glucose measurements were performed in infants who did not meet criteria as SGA/LGA per unit guidelines for that time period. It is possible that subjective assessment by nursing or medical personnel of abnormal growth led to blood glucose measurements outside of unit guidelines. It is unclear how this may have biased results. Inaccurate pregnancy dating is an additional confounder. Last menstrual period alone was utilized for dating when first-trimester ultrasound biometry was not available, which may be unreliable. Furthermore, as a single center, our results may not be generalizable to other delivery populations. However, the study population is heterogeneous reflecting the racially, ethnically, and economically diverse population served by the medical center. Our study only evaluated two commonly utilized growth standards. Inclusion of additional growth standards or absolute weight cut-offs may have identified a better-performing weight and/or weight-for-age guideline for hypoglycemia screening.

In conclusion, despite recommendations to screen SGA/LGA infants for hypoglycemia, there is no consensus on the optimal growth classification standard in the term well nursery population. Our comparison of FGC and WGC demonstrated poor agreement in classification with the potential consequence of suboptimal screening for hypoglycemia. The WGC was non-inferior to the FGC for relative sensitivity, PPV and NPV and was more sensitive in detecting infants with hypoglycemia, severe hypoglycemia, and hypoglycemia requiring intervention. Well baby nurseries should consider adopting the WGC to better identify infants at risk for hypoglycemia due to SGA/LGA status.

## Supplementary information

Supplementary Figure 1: Well Baby Hypoglycemia Protocol

Supplementary Figure 2: Distribution of Birth Weights

Supplementary Material Titles

Supplemental Material Summary

## Data Availability

The authors will make available a deidentified dataset at the request of readers.
