# Supplementary figures and images for: The performance of growth charts in well term newborns in screening for hypoglycemia
Source: J Perinatol. 2025 Aug 5;45(10):1352–9. doi: 10.1038/s41372-025-02373-3 (PMC12479346; doi:10.1038/s41372-025-02373-3)

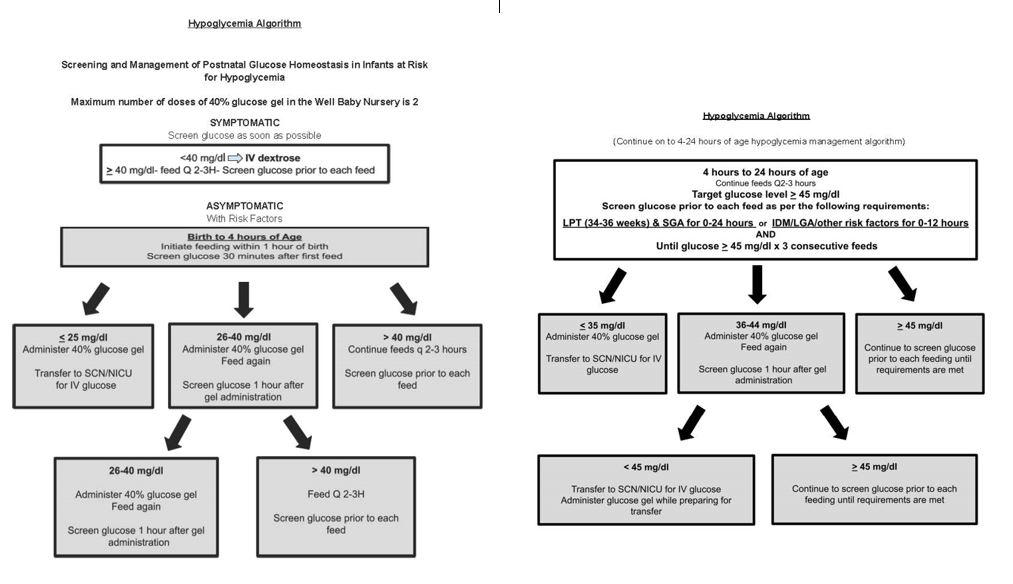

Supplement: Supplementary file 1 — Supplementary Figure 1: Well Baby Hypoglycemia Protocol [file 41372_2025_2373_MOESM1_ESM.jpg]

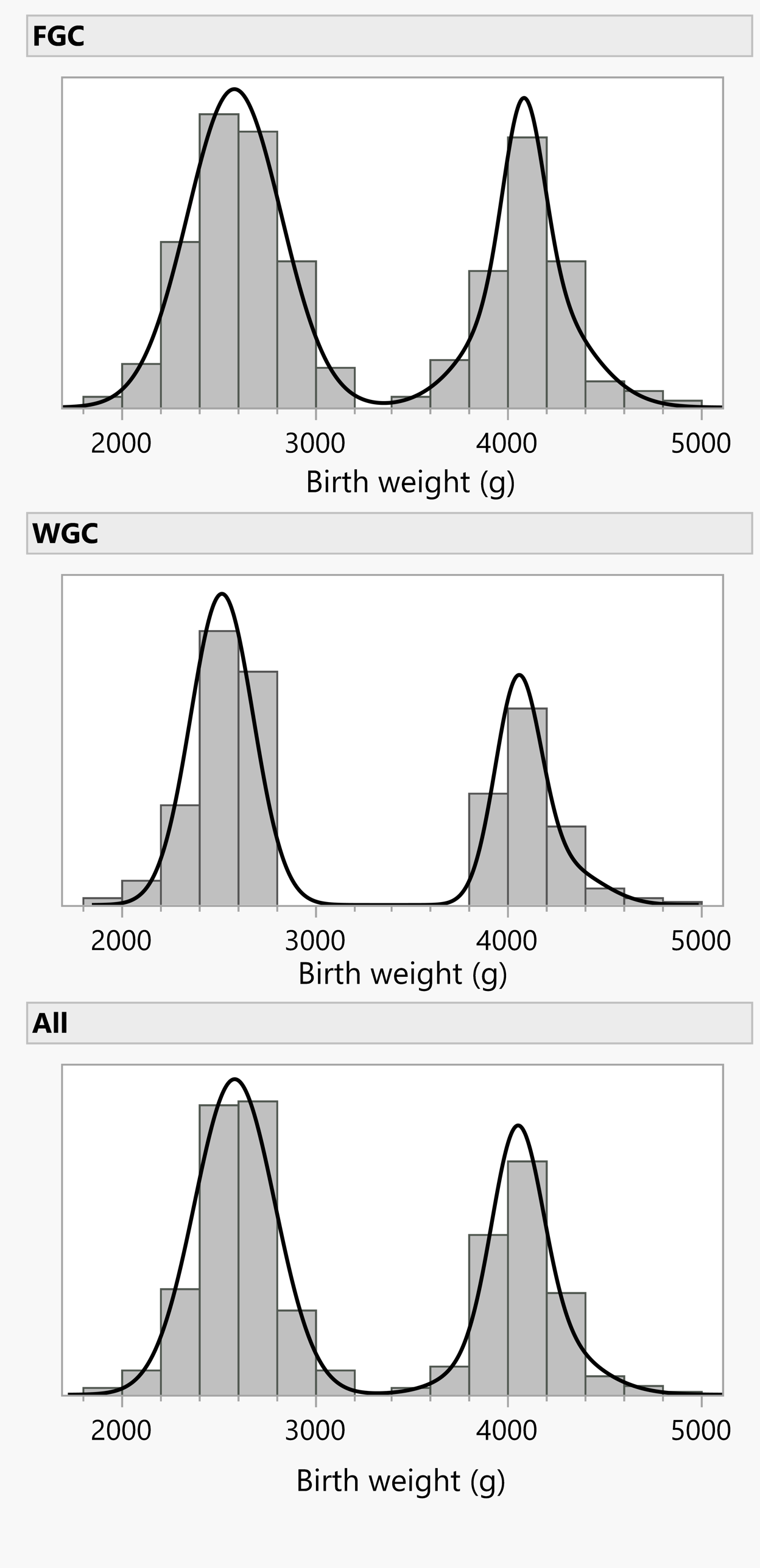

Supplement: Supplementary file 2 — Supplementary Figure 2: Distribution of Birth Weights [file 41372_2025_2373_MOESM2_ESM.png]
