# Supplementary material for: The performance of growth charts in well term newborns in screening for hypoglycemia
Source: J Perinatol. 2025 Aug 5;45(10):1352–9. doi: 10.1038/s41372-025-02373-3 (PMC12479346; doi:10.1038/s41372-025-02373-3)
Supplement: Supplementary file 3 — Supplementary Material Titles [file 41372_2025_2373_MOESM3_ESM.docx]

Supplementary Figure 1 title: Well Nursery Hypoglycemia Management

Figure 1 Legend: NONE

Supplementary Figure 2 title: Distribution of Birth Weights

Figure 2 legend: Histogram of birth weights of infants classified by the Fenton Growth Chart (Top Panel), WHO Growth Chart (Middle Panel) and the entire study cohort (Bottom Panel) as SGA or LGA. Only infants with blood glucoses available were included in the study population. Infants of diabetic mothers were excluded.
