# Supplementary material for: The performance of growth charts in well term newborns in screening for hypoglycemia
Source: J Perinatol. 2025 Aug 5;45(10):1352–9. doi: 10.1038/s41372-025-02373-3 (PMC12479346; doi:10.1038/s41372-025-02373-3)
Supplement: Supplementary file 4 — Supplemental Material Summary [file 41372_2025_2373_MOESM4_ESM.docx]

**Supplementary Material Summary:**

**Supplementary Figure 1: Well Baby Hypoglycemia Protocol**

This figure includes the protocol for the screening and management of infants at risk for hypoglycemia at Joseph M. Sanzari Children’s Hospital at Hackensack University Medical Center between 2021-2023.

**Supplementary Figure 2: Distribution of Birth Weights**

This figure depicts the distribution of birth weights for infants classified as SGA or LGA by Fenton Growth Chart, WHO Growth Chart and All SGA/LGA infants.
